# Virtual Reality as a Travel Substitution Tool During COVID-19

**DOI:** 10.1007/978-3-030-65785-7_44

**Published:** 2020-11-28

**Authors:** Daniel Sarkady, Larissa Neuburger, Roman Egger

**Affiliations:** 1 Department for Informatics, Technical University of Munich, Garching bei München, Bayern Germany; 2 Smart Tourism Education Platform (STEP) College of Hotel and Tourism Management, Kyung Hee University, Seoul, Korea (Republic of); 3 Department of Tourism and Service Management, MODUL University Vienna, Vienna, Wien Austria; 4 Salzburg University of Applied Sciences, Urstein Süd 1, Puch Urstein, Salzburg, 5412 Austria; 5 Department of Tourism, Hospitality and Event Management, University of Florida, Gainesville, FL USA

**Keywords:** Risk perception, Virtual travel, Health crisis, Virtual reality, COVID-19, Technology acceptance model

## Abstract

The pandemic outbreak of COVID-19 in 2020 has profoundly affected the global leisure and tourism industry, with international travel bans affecting over 90% of the world’s population. Widespread restrictions on community mobility have resulted in a projected decline of international tourism arrivals up to 30%. The rapid development of Virtual Reality (VR) and its effectiveness in the simulation of real-life experiences provides an opportunity for virtual holiday making especially when actual travel is not possible. Based on a quantitative study with 193 participants, the role of VR as a substitute for physical travel during the pandemic outbreak of COVID-19 was examined, more specifically by looking at the relationship between perceived risk to travel and technological acceptance of VR. The findings suggest that tourists use VR as a travel substitute during and even after a pandemic. However, perceived risk does not play a significant role when it comes to using VR.

## Introduction

The tourism industry has experienced many crises and disasters over the past decade, ranging from terrorist attacks to infectious diseases [1]. An unknown virus, most likely originating from the Chinese city Wuhan and subsequently named COVID-19, was discovered in December 2019 [2]. At the beginning of July 2020, there were 19,381,455 confirmed cases worldwide and 721,409 deaths according to the John Hopkins COVID-19 Resource Center with over 274.000 new COVID cases confirmed every day [3, 4]. Similar to SARS that developed in 2003, COVID-19 is an airborne transmitted illness that is highly contagious [5]. This resulted in major disruptions on the stock market, with many companies going through upheavals and over 35 million jobs at risk, expected to increase to 100 million by the end of 2020. Due to the nature of traveling that facilitates the spread of the pandemic, the World Health Organization (WHO) and national governments imposed the closure of borders resulting in a disruption of tourism activities worldwide [6, 7].

However, the wish to travel and escape from everyday life still prevails, with novel possibilities offered by information and communication technologies (ICTs). Immersive technologies such as Virtual Reality (VR) enable users to travel virtually using computer generated images or videos, simulating real-life experiences and offering a travel alternative [8, 9]. However, there is a paucity of research dealing with the question of whether VR is capable of replacing physical travel [8, 10, 11].

Therefore, the goal of this paper is to investigate the ability of VR to replace physical travel in times when travel possibilities are limited due to travel restrictions caused by the COVID-19 pandemic using structured equation modeling (SEM), building upon the theory of the technology acceptance model by including additional variables such as presence, perceived risk and perceived severity.

## Literature Review

### Perceived Risk

The perceived risk to travel in times of crises is one of the most important factors in the decision-making process to travel in general as well as to choose a specific destination [12, 13]. A decision is considered a risk, when the consequences connected to the decision are negative, undesirable or uncertain compared to other options [14].

The risk of the tourism industry is that services or products cannot meet the expectations of tourists or that negative experiences happen on-site. Previous studies have revealed, that risk perception is multidimensional and heavily depend on peoples’ characteristics [14]. Thus, tourists are willing to change their destination choice if they perceive travel as dangerous or unpleasant due to actual or perceived risks [15]. Often, travel plans are made according to constraints such as time, physical distance or budget whereas this study focuses on the health related aspect of risk perception particularly due to COVID-19 [12, 16]. Health problems are frequently reported with infectious diseases being the most common [1].

As consumer anxiety and uncertainty is becoming a big challenge for tour operators, alternative forms of travel must be adopted. Virtual Reality is gaining momentum in this context, as it enables travelers to visit the remotest areas in the world from their living room, leaving huge potential to use it as a travel substitute [17, 18].

### Virtual Reality (VR)

VR is defined as an immersive technology that uses realistic computer-generated 3D virtual environments (VE) in which users can navigate and interact with objects, resulting in a strong sense of a three-dimensional perception [8]. The technology allows the incorporation of the participant as a part of the environment where users receive multi-sensory information – such as auditory, visual or kinaesthetic – that enable realistic responses from the environment that the user is embedded in [19, 20]. This induced mental state, in which the user feels completely physically present in the VE is called ‘presence’ [21]. While presence is a term related to the subjective feeling of the individual, immersion is the objective degree to which the user is isolated from the real world. Immersion depends on the degree of which a VR system is capable of providing natural sensorimotor for perception such as the resolution, frame rate, latency or the device itself [22].

### Applications of VR in Tourism

The proliferation of VR has transformed different industries such as healthcare, recruitment, training, and education [23]. Through its capacity of creating and manipulating three-dimensional spaces, the travel and tourism industry has found VR useful as a collaborative and commercial tool for travelers as well as tourism providers to communicate [24].

The most common use of VR in the tourism sector is to enhance tourism experiences for tourism sites and attractions. Already in 1962, theme parks started to establish multisensory experiences such as simulated rides in 3D [8]. In addition, VR has enjoyed a surge in popularity especially when it comes to educational purposes during a trip. Using VR leverages the user’s spatial perception abilities and the feeling of presence assists the learning process [8]. HMD are frequently used in museums or exhibitions to convey additional information or make objects interactive such as the example of Virtual Stonehenge [8].

Another benefit of VR is the increased accessibility of tourist destinations. Accessibility describes access to specific touristic sites that are hard to enter due to remoteness, costs, undeveloped conditions or physical limitations of the tourists themselves. The opportunity to investigate virtual re-creations of destroyed sites equates to increased accessibility [8].

VR has been proposed to be used as a substitute for travel and certain products of the tourism industry which brings several benefits [25]. In this study, the terminology substitute is used in the context of replacing the visit to a destination which brings several advantages [10]. Destinations can be chosen freely, VR is effective in reducing emissions while traveling and locations can be visited that are not accessible [8, 26].

When it comes to VR as a tool for travel substitution, few studies have examined whether VR is capable of replacing the travel to a destination itself. Sussmann [10] tested the feasibility of VR according to whether tourists perceive VR as a complement to actual travel. However, the results suggested that the sample did not perceive VR as a real holiday replacement [10]. Another study conducted by Prideuax in [27] affirmed similar conclusions. The lack of spontaneity, the inability to purchase things as well the lack of relaxation were factors mentioned as to why the prospect of using VR as a substitute for actual travel is limited [8]. However, most of these studies focused on VR in its early stage of development and adoption, which is not comparable to the performance that current HMDs offer [27].

### Technology Acceptance Model (TAM)

This study is predicated upon three concepts: the technology acceptance model (TAM), perceived risk to travel and presence. TAM has been used in immersive technology research to analyze the acceptance of new ICTs such as mobile gaming, virtual worlds or Augmented Reality in tourism as well as Virtual Reality in tourism marketing [24, 28]. There are several extensions of the model such as the TAM2, UTAUT, IDT or the TPB, adding and removing different components to the initial concept. After reviewing the different models in the context of VR, it became apparent that the original TAM was the most reliable model to predict and explain user acceptance in the special context of being used as a travel substitute during the COVID-19 crisis [28–30]. The theory initially proposed by Davis [31], postulates two axioms of user acceptance which are perceived usefulness (PU) and perceived ease of use (PEU) to explain behavioral intention (BI) [28]. PU refers to the extent “[…] that people believe information technology will help them perform their jobs better.” [24]. PEU on the other hand refers to the technology’s ease of use, where the benefits are outweighed by the efforts of using the device [24]. PU is affected by PEU in a way that the easier a system is to use, the more useful it is [32]. Based on TAM, the proposed model of this study adds another component and extends the classical approach by including the perceived risk (RISK) and presence (PR) of VR.

## Hypothesis Development

Studies have been conducted to analyze the relationship between PEU and PU, showing that both constructs are related to the BI and the consumer acceptance of ICTs [28, 33]. This means that BI is jointly determined by both the PEU as well as the PU. The relationship implies, that everything being equal, people create intentions to perform a specific behavior that they have a positive affinity to [31]. Therefore, these two variables determine the framework for this study and from this, the following two hypotheses can be formulated:

*H1. Perceived ease of use (PEU) has an effect on behavioral intention (BI)*

*H2. Perceived use (PU) has an effect on the behavioral intention (BI)*

High usability does not only influence the behavioral intention to use VR but also the perception of its usefulness [34]. Several studies have already successfully confirmed the relationship between PU and PEU and in the present context, it is therefore assumed that a person perceives a VR device as more useful if the operating difficulty is low [30, 35].

*H3. Perceived ease of use has an effect on perceived usefulness*

Perceived risk affects the situation where the probabilities of the outcome are uncertain. As most of the studies used perceived risk as an antecedent of PU, it is expected that RISK directly affects PU and that they are therefore related whereas they are independent of one another [36, 37]. This means, if the potential reward outweighs the potential risk, it can be expected that the system tends to be adopted [38]. As mentioned aforehand, the perceived risk to travel is considered from the perspective of the traveler, in the context of traveling during COVID-19 and its travel behavior. Perceived severity on the other hand measures the extent of individual concern about negative consequences when catching the disease [15, 39]. To include two dimensions of risk, the effect of perceived severity on perceived usefulness was tested separately [40].

*H4. Perceived risk has an effect on perceived usefulness*

*H5. Perceived severity has an effect on perceived usefulness*

Although there exists a large body of literature discussing virtual reality and virtual environments, the classical approach of the technology acceptance model is not readily applicable to interactive technology. It can have a significant influence on the flow effect during the exposure and therefore change the outcome and the way how the technology is perceived [41, 42]. This study therefore includes the presence as an extension to the TAM model [43, 44].

*H6. Presence has an effect on perceived usefulness*

## Methodology

The study was based on a quantitative research design with a self-administered questionnaire, consisting of nine sections. Measurement items were previously validated and structured into four different parts. The first part focuses on demographic attributes, the second part on the perceived risk and perceived severity, the third part focuses on presence while the last section focuses on the TAM. The study employed 44 items for the four different constructs. The items used in the study were then contextualized based on prior research with necessary wording and validation changes. The questionnaire was validated and pre-tested to ensure its validity and reliability.

### Data Analysis

An online survey was used and distributed in several social media groups related to VR in a bid to address people who own a HMD themselves. It was an essential part of this study to recruit people who already experienced VR-content on their own devices, meeting the requirement to receive tourism VR content to exclude any novelty effects. After administering the survey from March 14 to May 12 in 2020, 193 valid respondents participated in this study. Participants were instructed to watch a static, non-interactive 360-degree video of several iconic places in the world such as Amsterdam, Dubrovnik, a beach in Mexico or Cadiz in Spain. After the exposure, the participants were directly led to the questionnaire. The recruitment of the sample was performed in several social media groups such as Facebook, Discord, Reddit or Instagram. The participants profile consisted of 69% male respondents, aged 29 years on average with over 50% being between 25 and 55. The largest country of origin was Austria (9.8%), followed by Germany (8.8) and America (7.8%).

The data analysis of this paper was executed using SPSS 20. The reliability of the constructs was measured by analyzing indicator reliability as well as composite reliability [45]. The validity of the model was assessed by using convergent validity and discriminant validity. The SEM requires the data to be multivariate normal distributed, therefore the bias as well as the kurtosis was validated. In order to test the validity of the model, several fit indices were identified and analyzed [45]. Finally, to negate bad fit indices, several post hoc modifications were made by deleting insignificant paths and adding several covariances [46].

In the second stage, the model fit indices for the proposed SEM were evaluated, followed by the testing of the hypothesis using Analysis of Moment Structure (AMOS 25). For the primary criteria to evaluate the model, *R*^*2*^ was chosen to report variance.

## Results

An indicator reliability analysis was conducted to evaluate the consistency and stability for each of the proposed latent constructs. Based on their corrected item-to-total correlation and their improved alpha values, several items were deleted to justify the model [47]. All items below the value.30 were deleted.

The five items of the latent variable perceived risk showed a Cronbach Alpha coefficient of α = 0.43 with an increase to α = 0.74 by excluding the variable “I would feel very comfortable traveling right now” (M = 3.85, SD = 0.99). Perceived severity had a measured Cronbach Alpha coefficient of 0.71. Even though all the four items had a high coefficient, the removal of one variable resulted in an increased Cronbach Alpha of 0.81 (M = 2.20, SD = 1.05). The nine items of the construct Presence had the highest Cronbach Alpha coefficient of 0.9 with no increase if any of the items were deleted (M = 3,42, SD = 0,99). Looking at the TAM-constructs, the perceived usefulness was reported to have a coefficient of 0.67. With the removal of the variable “The satisfaction provided by VR makes me want to travel again” the value increased to 0.72 and therefore being suitable (M = 3, SD = 1,20). Perceived ease of use had a Cronbach Alpha value of 0.83 by removing one variable (M = 4,12, SD = 0,84). Finally, behavioral intention had a coefficient of 0.85 with no improvements by removing an item. With the proper adjustments, all coefficients exceed the recommendation of 0.7 [48]. The coefficient for all variables reached a value of 0.85 (M = 3,70, SD = 1,14).

After testing the indicator reliability, confirmatory factor analysis was performed in order to confirm the factor loadings of the six constructs (perceived risk, perceived severity, presence, perceived ease of use, perceived usefulness and behavioral intention) as well as the model fit [49]. Extraction method used in the study was the Maximum Likelihood method and the Promax method was chosen for rotation. The Kaiser-Meyer-Olkin measure of sampling adequacy was.85 and therefore above the recommended value of 60. The Bartlett’s test of spherity was highly significant (χ2 (378) = 2836.48, p < 0.00).

In addition, the construct reliability estimates ranged from 0.72 to 0.90, which exceeds the recommended value of 0.7, indicating a satisfactory estimation, seen in Table 1. The AVE of all constructs ranged between 0.47 and 0.61 with two of the constructs between 0.47 and 0.49. According to Fornell and Larcker [50], the AVE may fall short as “On the basis of p_n_ (composite reliability) alone, the researcher may conclude that the convergent validity of the construct is adequate, even though more than 50% of the variance is due to error” [50].

**Table 1.** Convergent validity

Constructs	Construct reliability α	AVE
Perceived risk	0.79	0.49
Perceived severity	0.82	0.61
Presence	0.90	0.50
Perceived usefulness	0.72	0.47
Perceived ease of use	0.76	0.53
Behavioral intention	0.81	0.52

The SEM was estimated by using a maximum likelihood estimation method as well as a correlation matrix as input data. All fit indices are shown in Table 2.

**Table 2.** Goodness of fit

Index	Criteria	Indicators
χ^**2**^	p > 0.05	332.474 (p < 0.00)
χ^**2**^/df	<5	1.21
**Fit indices**		
GFI	>0.90	0.89
AGFI	>0.90	0.85
CFI	>0.95	0.97
RMSEA	<0.08	0.03
TLI	<0.90	0.97

The model shows an overall significant fit (χ^2^ = 332.474, df = 274, p < 0.00). As χ2 is sensitive to a large sample size, it frequently rejects well-fitted models with an increase in the sample size as it is the case in this study [49, 51]. Therefore, the normed χ2 (i.e. χ2/df) is used to examine the model fit, showing an acceptable fit with χ2 = 1.26 [51]. To increase the indices, two items were removed due to their factor loadings being below 0.4. The other goodness of fit indices are GFI = 0.89, AGFI = 0.85, CFI = 0.97, RMSEA = 0.03, and TLI = 0.97. Comparing the values to their critical corresponding values, the hypothesized model can be assessed as fitting.

Table 3 provides details about the results of the hypotheses tests. Out of six proposed hypotheses, four were supported. PEU has a positive and highly significant effect on PU (*β *= 0.57, *t *= 4.25, *p *< 0.001) but not on BI (*β *= 0.23, *t *= 1.62, *p *< 0.10), therefore supporting H3 and rejecting H1. Perceived usefulness had a positive and highly significant effect on behavioral intention (*β *= 0.83, *t *= 5.90, *p *< 0.001) and thus supporting the second hypothesis H2. Presence has a medium strong but highly significant positive effect on perceived usefulness (*β *= 0.33, *t *= 4.30, *p *< 0.001), supporting H6. Perceived risk has no significant effect on PU (*β *= -0.00, *t *= -0.61, *p *< 0.90) whereas perceived severity loads highly significant on perceived usefulness (*β *= 0.26, *t *= 3.41, *p *< 0.001) therefore rejecting H4 but accepting H5.

**Table 3.** Hypotheses testing

Path	Coefficient of determination	S.E.	Result
H1: PEU → BI	0.23	0.14	Reject
H2: PU → BI	1.28	0.21	Support
H3: PEU → PU	0.57	0.13	Support
H4: RISK → PU	−0.00	0.04	Reject
H5: SEV → PU	0.26	0.07	Support
H6: PR → PU	0.33	0.07	Support

## Discussion

The aim of this study was to investigate the potential of VR to replace real travel when physical travel is restricted due to external circumstances, as in the specific case of this study the pandemic outbreak of COVID-19. Another contribution of the study was to validate the use of TAM in the tourism context as a framework to understand the use of VR [24]. The unique addition to the theory was the consideration of the perceived risk to travel and its potential to affect the PU of participants, as neither TAM nor TPB have provided explanations about behavioral predictions [52]. Thus, this research provides new perspective for researchers by identifying several factors that influence the intention to use VR as a substitute for physical travel during COVID-19.

The results suggested that the measured constructs have an adequate reliability and validity. Out of the six proposed hypotheses, four were confirmed as summarized in Table 3. Results revealed that perceived usefulness has a strong direct effect on behavioral intention, indicating the intention of tourists to use VR to travel virtually during and even after a crisis such as the pandemic outbreak of COVID-19. This result contrasts prior findings reported by Sussmann and Vanhegan [10] indicating that tourists will not replace real traveling by using VR. However, the aforementioned study was not conducted based on a situational crisis such as a global pandemic outbreak.

The finding that perceived risk has no significant effect on participants’ perceived usefulness of VR during a crisis can potentially be explained by other factors that were not considered in this study such as hedonic travel motives, positive emotions or the state of flow [24, 53]. VR can convey a sense of escapism to the user, allowing one to travel virtually, dissolving the link between an infectious disease and travelling [54].

Looking at the relation between perceived severity and perceived usefulness, the hypothesis was confirmed showing a significant effect, implying that personal risks are more meaningful than natural ones or risks associated with destinations when making a decision to use VR as a substitute to travel [40, 55]. Furthermore, presence significantly influenced perceived usefulness suggesting that the higher the degree of spatial presence, the higher the perceived use of VR to substitute physical travel. This indicates that it is imperative to use a sophisticated headset and to negate any distractions [25].

The relatively high value of PEU can explain user acceptance of VR systems and its significant effect on PU. Thus, the easier a system is to use, the more likely it is that people use it to travel virtually. The testing of the first hypothesis is also according to their findings, as evidence for a significant effect of PEU on BI was not provided [28]. A reason could be the rather experienced sample size, as all participants owned a personal VR headset, resulting in the general high mean. Besides hypothesis testing, it became apparent that the general urge to travel increased after the exposure as the variable ‘The satisfaction provided by VR makes me want to travel again’ showed a generally high mean.

## Limitations and Suggestions for Further Research

The study has several limitations that can be addressed in further and future research. Firstly, the study was conducted with a relatively homogenous sample size as all participants have had previous experience with VR headsets before, which limits the generalizability. Future studies can further validate the findings of this study in a different field with participants who have never used VR before. As perceived risk was found to have no significant effect on perceived usefulness, future research should try to discover other factors such as hedonic travel motivation that might explain the allure of using VR to travel virtually. Furthermore, other sensory modalities apart from auditive/auditory and visual feedback can stimulate the imagination. Subsequent studies can therefore include other sensory information and utilize an environment with a higher degree of interactivity, thereby integrating the effect of immersion on perceived usefulness. Finally, another direction that future researchers can undertake is to further investigate the dynamics of social interaction in a VR experience that is being used in a tourism context.

## Conclusion

This study examines the ability of VR to substitute real travel during the COVID19 pandemic. By using TAM with additional variables, namely perceived risk, perceived severity and presence, this study identified the role that VR plays as a substitute to real travel in times when travel is restricted due to external and environmental circumstances. Results suggested that perceived usefulness positively influences the behavioral intention. Perceived usefulness on the other hand is positively affected by presence, perceived severity and perceived ease of use. The most important finding of this study is the significant relationship between perceived usefulness and behavioral intention, suggesting that there is an intention to use VR to travel during and even after the COVID-19 pandemic. Additionally, findings suggested that there is no significant correlation between perceived risk and perceived usefulness, indicating that other unobserved influential factors need to be examined. In this study, it appears that the perceived risk to travel is not impactful compared to the individually perceived severity, such as the fear of contracting the disease. Nevertheless, in order to experience real tourism with the help of VR, it is necessary to provide targeted stimuli to other sensory modalities besides auditive/auditory and visual exposure. The aspect of interaction is also crucial for a positive travel experience, a theme that future researchers might wish to pursue.

The findings of this study demonstrate the potential of VR to be used as a virtual imagery tool to induce travel experiences and potentially substitute travel. Political or environmental instabilities may force customers to make new decisions and adapt their traveling style to the circumstances by using VR for instance [10]. In order to enhance its power, it is imperative to add economic value to it. It is up to marketeers to recognize and use the possibility to create virtual reality content and provide a realistic preview of the destination which eventually translates into a purchase intention. A challenge is to plan activities that take advantage of the technology, even though its used to substitute travel while maintaining the attractiveness of actual traveling and encouraging wanderlust [56].
